# Micro-CT Evaluation of Screw Deformation After Quasi-Static Loading of Titanium-Base and Multi-Unit Abutments at Different Implant Angulations

**DOI:** 10.1155/ijod/2519930

**Published:** 2025-06-13

**Authors:** Kübra Nur Duran Ilhan, Mehmet Erdem Ilhan, Gülsüm Sayın Ozel

**Affiliations:** ^1^Department of Prosthodontics, Institute of Health Sciences, Istanbul Medipol University, Istanbul, Türkiye; ^2^Department of Prosthodontics, Faculty of Dentistry, Istanbul Medipol University, Istanbul, Türkiye

**Keywords:** load-bearing capacity, micro-CT, multi-unit abutment, prosthetic screw, screw bending, SEM, Ti-base abutment

## Abstract

**Objectives:** The aim of this research is to determine the effects of the variations in the implant angulation and the selection of abutment type on the mechanical potential of prosthetic components and to compare the deformations of abutment screws through micro-CT analysis.

**Materials and Methods:** Forty titanium implants, each measuring 4.1 mm in diameter and 12 mm in length, were positioned in acrylic resin models in both straight and angled positions. The specimens were classified into four groups (*n* = 10): (1) straight-placed implant with titanium-base (Ti-base) abutment crown (Ti-1), (2) angulated-placed implant with Ti-base abutment crown (Ti-2), (3) straight-placed implant with multi-unit abutment crown (MU-1), and (4) angulated-placed implant with multi-unit abutment crown (MU-2). All groups underwent thermal aging (5,000 cycles, 5°C–50°C, 30-s dwell time) in distilled water for 24 h. A quasi-static load was applied to each specimen utilising a universal testing machine, and the highest force values at deformation were documented. The failure mode was ascertained by micro-CT and scanning electron microscopy (SEM) imaging.

**Results:** The mean failure load values observed in the study were 2589 N for group Ti-1, 1768 N for group Ti-2, 3318 N for group MU-1, and 2213 N for group MU-2. Group MU-1 exhibited a higher failure load value than group Ti-1, Ti-2, and MU-2. Screw bending was observed in all groups. Groups Ti-2, MU-1, and MU-2 showed a significant difference in the change of angle values on the abutment screws, while group Ti-1 did not show any statistical difference (*p*=0.001; *p*  < 0.05).

**Conclusion:** The type of abutment and the angle of implant placement can affect the maximum load capacity of the implant–abutment complex. Both Ti-base and multi-unit abutments exhibited notable resistance forces.

## 1. Introduction

The dental implant treatment is a popular choice in many cases due to its impressive success rate [1, 2]. Various options exist for restoring implant-supported prostheses, suitable for both screw- and cement-retained applications, each with distinct advantages and disadvantages in clinical practice [3]. Recently, the benefits of screw-retained restorations, particularly concerning retrievability and maintenance, have made them more favorable than other options. Cement-retained restorations carry a risk of implant failure due to biological issues, such as peri-implantitis caused by subgingival cement residue. For these reasons, screw-retained restorations are currently the preferred choice, offering superior biocompatibility and retrievability [4–7].

There are various abutment options for achieving screw-retained implant prostheses, including Ti-base abutments and multi-unit abutments. Titanium-base (Ti-base) abutments have proven to be successful and show higher survival rates, making them a popular choice in modern implant dentistry [8, 9]. Furthermore, Ti-base abutments were developed to provide a more appealing abutment option while leveraging the advantages of the connection between titanium components [10]. Titanium is the preferred metal for dental implants and materials, a preference driven by several key factors, including its superior material toughness, crack resistance, and excellent anticorrosive properties [11]. Additionally, personalized zirconia abutments or crowns can be created through CAD–CAM technology and are often paired with prefabricated titanium abutments for esthetically pleasing dental restorations [12]. This design, known as the hybrid-abutment concept, includes a Ti-base abutment that supports all esthetic crowns [13, 14].

A multi-unit abutment is an intermediary component used to join multiple implants, enabling screw-retained implant restoration. Commonly utilized in full-mouth implant rehabilitation protocols, multi-unit abutments are available in both straight and angled versions (17°, 30°, and 45°) for use with angled implants in partially or completely edentulous patients. When multiple implants are connected to a screw-retained implant restoration, straight and angled multi-unit abutments provide a common insertion path, compensating for variations in implant angulation [15]. Screw-retained prostheses designed for multiple implant cases are attached to a milled titanium cap, or sleeve, which is subsequently screwed onto the multi-unit abutment [16–18].

The multi-unit abutment system is configured as a dual-screw system, whereas the Ti-base abutment system employs a singular screw to attach the prosthetic restoration to the implant. A mini-screw fastens the prosthesis to the multi-unit abutment following its attachment to the implant in the multi-unit system. The manufacturer supplies a titanium sleeve, or cap, that is attached to the prosthesis surface to ensure an appropriate fit on the multi-unit abutment. The design variations among different abutment types can profoundly influence their long-term efficacy in supporting the prosthesis.

Implant-supported fixed restorations are growing increasingly popular due to a heightened focus on esthetic results, developments in materials, and the necessity for reversibility in implant-supported prosthetics. The appropriate selection of prosthetic components is essential for a successful and sustainable implant-supported restoration [19]. Although numerous studies evaluate the durability of CAD–CAM manufactured crowns with Ti-base abutments and the associated cementation methods, the number of studies contrasting the force resistance of Ti-base and multi-unit abutment screws is still limited. This experimental study was designed to assess the mechanical performance of prosthetic screws with two distinct abutment types at maximum load in quasi-static settings. The null hypothesis posited that no statistically significant differences would exist between Ti-base and multi-unit abutments regarding the mechanical resistance of the prosthetic screw.

## 2. Materials and Methods

**Figure 1:**
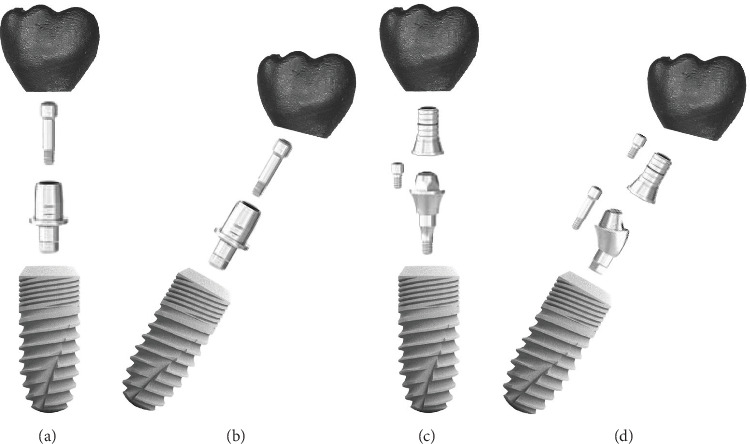
Prosthetic components of the study specimens. (a) Ti-base 1, (b) Ti-base 2, (c) multi-unit 1, and (d) multi-unit 2.

A power analysis was conducted prior to the study to determine the required sample size. Using G-Power software (version 3.1.9.6) with an estimated medium effect size of 0.25 and with 85% power and a significance level of 0.05 [20]. The overall specimen size was determined to be 42. Forty implants (T6 Standard, NucleOSS, Turkey), each measuring 4.1 mm in diameter and 12 mm in length, were examined. The implants were divided into four groups (*n* = 10 each) based on abutment type and implant angulation. All groups used screw-retained abutments restored with crowns made of cobalt-chromium alloy (Figure 1).

The study groups were as follows:

 
**Ti-base 1 (Ti-1):** a straight implant-supported screw-retained full metal crown adhered to a prefabricated Ti-base abutment (CAD–CAM hex Ti-base abutment, 4.5 mm diameter, 0.7 mm gingival height; NucleOSS, Turkey).

 
**Ti-base 2 (Ti-2):** an angulated (30°) implant-supported screw-retained full metal crown adhered to a prefabricated Ti-base abutment (CAD–CAM hex Ti-base abutment, diameter 4.5 mm, gingival height 0.7 mm; NucleOSS, Turkey).

 
**Multi-unit 1 (MU-1):** a straight implant-supported screw-retained full metal crown attached on multi-unit abutment (multi-unit abutment [straight], with a diameter of 5 mm, gingival height of 1.5 mm; NucleOSS, Turkey).

 
**Multi-unit 2 (MU-2):** an angulated (30°) implant-supported screw-retained full metal crown attached on multi-unit abutment (multi-unit abutment [17°], diameter 5 mm, gingival height 1.5 mm; NucleOSS, Turkey).

### 2.1. Fabrication of Specimens

The specimen models were fabricated using self-curing acrylic resin (Imıcryl, Turkey), which was poured into cylindrical molds produced by a 3D printer (Creality Ender-3 V2, China). To represent the mandibular first molar, implants were positioned in both straight and angled orientations within the models. The surgical kit (T6 Standart, NucleOSS, Turkey) was used in conjunction with a parallelometer (Amann Girrbach AF 350) to ensure standardized implant positioning.

Ti-base and multi-unit abutments were attached to the implants placed in the acrylic models and grouped accordingly. Scan bodies (NucleOSS) were placed on the Ti-1, Ti-2, and MU-1, MU-2 groups and scanned using a laboratory scanner (Identica, MEDIT, Seoul, South Korea). The digital files were imported into CAD software (ColLab Scan v.2.0.0.3) for crown design. Screw-retained, full-contour metal crowns were designed with dimensions of 9 mm in length and 10 mm in width, replicating the mandibular first molar. This design was consistently reproduced to maintain standardization across all groups. All restorations were milled from CAD–CAM wax blanks (Al Dente, Germany) and cast in traditional Cr–Co alloy (Keralloy N, Siladent, Germany).

The crowns were attached to the Ti-base abutments and multi-unit abutments using commonly recognized methods. Due to design differences between the two types of abutments, the connection method to the crowns and the torque procedures varied.

### 2.2. Ti-Base Groups Cementation

The internal surfaces of the crowns and the external surfaces of the Ti-base abutments (groups Ti-1 and Ti-2) were sandblasted with 50 μm aluminum oxide particles (Cobra, Renfert, Hilzingen, Germany) using a blasting pressure of 2.5 bars for 20 s at a distance of 10 mm. After sandblasting with aluminum oxide particles, the abutments and crowns were ultrasonically cleaned in deionized water for 15 min. The crowns were then cemented to the Ti-base abutments using self-curing, resin-based cement (multilink hybrid abutment cement, Ivoclar Vivadent), following the manufacturer's instructions.

A thin layer of cement was applied to the Ti-base surfaces and the intaglio surfaces of the crowns using a syringe, and the Ti-base abutments and crowns were initially seated with finger pressure. Residual cement at the margins was removed with a disposable brush. Polymerization was conducted for 3 min using a high-intensity polymerization device (Woodpecker LED.G). Following cementation, the abutments and crowns in groups Ti-1 and Ti-2 were screwed to the implants with a torque wrench driver (NucleOSS, Turkey) to a tightening force of 30 Ncm. The screws were retightened after a 10-min interval to prevent potential loosening.

### 2.3. Multi-Unit Groups Cementation

The internal surfaces of the crowns and the external surfaces of the prefabricated titanium sleeve (Titanium cap, NucleOSS, Turkey) for multi-unit abutments (groups MU-1 and MU-2) were prepared using a sandblasting procedure with 50 μm aluminum oxide particles (Cobra, Renfert, Hilzingen, Germany) at a blasting pressure of 2.5 bars for 20 s at a distance of 10 mm. Following sandblasting, the titanium sleeves and crowns were ultrasonically cleaned in deionized water for 15 min. The crowns were then cemented onto the titanium sleeves using a self-curing, resin-based cement (multilink hybrid abutment cement, Ivoclar Vivadent), in accordance with the manufacturer's instructions.

A thin layer of cement was applied to the surfaces of the titanium sleeves and the intaglio surfaces of the crowns using a syringe, and the titanium sleeves and crowns were initially seated with finger pressure. Residual cement at the margins was removed with a disposable brush. Polymerization was performed for 3 min with a high-intensity polymerization device (Woodpecker LED.G). After cementation, the multi-unit abutments from groups MU-1 and MU-2 were screwed to the implants with a torque wrench driver (NucleOSS, Turkey) to a tightening force of 30 Ncm. The crowns were then placed onto the multi-unit abutments and tightened with a torque wrench driver (NucleOSS, Turkey) to a force of 20 Ncm. The screws were retightened after a 10-min interval to prevent potential loosening.

Each specimen's screw insert was coated with a layer of Teflon tape. The screw access channels were then filled with restorative material (Filltek Z250, 3M ESPE, USA) and photopolymerized for 60 s using a high-power polymerization device (Woodpecker LED.G).

### 2.4. Thermal Aging

A total of 5000 thermal cycles were performed in deionized water for 24 h, with temperatures ranging from 5°C to 55°C to simulate extreme thermal fluctuations and a dwell time of 30 s (SD Mechatronik Thermocycler, Westerham, Germany), simulating the intraoral conditions equivalent to approximately 6 months [21].

### 2.5. Static Loading Test and Failure Mode Analysis

To replicate the scenario of offset loading in a universal testing machine (AGS-X Universal, Shimadzu Corp), all specimens were subjected to quasi-static loading. The load was applied using a steel semi-spherical ball, with the loading point set at a 30° angle to the buccal cusp slope, 1 mm below the cusp tip, and a crosshead speed of 1 mm/min. A 0.3 mm-thick tin foil (Zinnfolie, Dentaurum, Ispringen, Germany) was placed between the crowns and the loading stamp to ensure uniform stress distribution. To achieve precise load application, the loading point was carefully aligned with each specimen by marking it according to the buccal cusp measurement. Additionally, all specimens were prepared to standardized dimensions and securely mounted in a consistent position within the specimen holder of the universal testing machine, ensuring uniform testing conditions across all samples.

Loading was applied continuously until either the specimens fractured or the testing device detected a reduction in force. The maximum force values at the point of deformation were recorded for each specimen.

A visual analysis was performed to determine the mode of failure for each specimen following the failure load test. The observed failure modes were classified as occurring at the crown, abutment, or screw level.

• Adhesive failure
• Crown deformation
• Implant deformation
• Abutment fracture
• Abutment bending
• Screw fracture
• Screw bending

### 2.6. Micro-CT Analysis

Specimens that failed due to metal deformation were examined using micro-CT imaging to determine whether bending or fracture had occurred in the screws or abutments. Each group was scanned with an X-ray microtomograph (MILabs U-CT, Netherlands) under the following settings: 88 kV source voltage, 0.43 mA source current, a 1 mm aluminum filter, an exposure time of 40 ms, and a rotation increment of 0.3°.

Image J software was employed to compare the micro-CT images of the Ti-1, Ti-2, MU-1, and MU-2 groups with those of their control counterparts, which consisted of intact screws that had not been subjected to loading. This comparison aimed to evaluate the degree of bending along the screw axes resulting from deformation under applied force.

The micro-CT images were further analyzed to assess angular changes in the screw axes by comparing two sets of angles. For the control group, angles were measured by identifying two points along the implant's long axis and a third point at the screw head tip on screws that had not undergone force application. In the study groups, these same reference points were used to measure screws that had been subjected to loading.

### 2.7. Scanning Electron Microscopy (SEM) Images

The most representative samples were analyzed using SEM (Gemini 500, Zeiss) to further assess the mode of failure. One specimen from each group was examined at magnifications of ×30, ×45, ×150, and ×500. For comparative purposes, the abutment and screw from intact specimens in each group were also imaged at the same magnifications. The SEM images provided detailed views of the cervical collar of the abutments and the screw threads, facilitating the interpretation of observed differences.

### 2.8. Statistical Analysis

The statistical analysis was performed using the Number Cruncher Statistical System (NCSS). The Shapiro–Wilk test was used to assess the distribution of the data. The Mann–Whitney *U* test was used to compare two groups. The Kruskal–Wallis test was utilized to compare differences across three or more groups. Statistical significance was determined at the *p* < 0.01 and *p* < 0.05 levels.

## 3. Results

**Figure 2:**
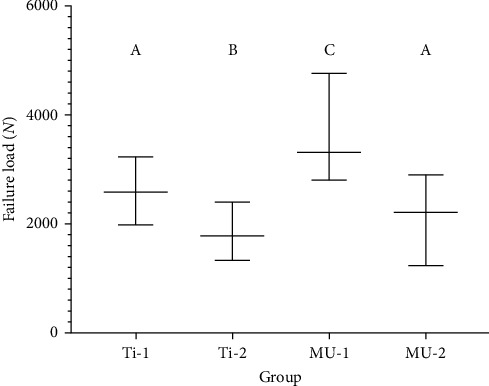
Representation of failure load (*N*) of the study groups after quasi-static loading. Different uppercase letters indicate significant differences among groups.

The results of this study demonstrate that all specimens successfully withstood the thermal aging protocol and were subsequently subjected to quasi-static loading. The force values (*N*) at the point of deformation varied depending on abutment type and implant angulation under offset loading. Group MU-1 exhibited a statistically higher load-to-failure value compared to groups Ti-1, Ti-2, and MU-2. Group Ti-1 showed a statistically higher load-to-failure value than groups Ti-2 and MU-2. Additionally, group MU-2 demonstrated a statistically higher load-to-failure value compared to group Ti-2. However, no statistically significant difference was observed between groups MU-2 and Ti-1. The parametric results are illustrated in Figure 2. The study indicated that the overall mean failure loads were 2589 N (Ti-1), 1768 N (Ti-2), 3318 N (MU-1), and 2213 N (MU-2). The findings from the quasi-static loading test, along with descriptive statistical analysis, are presented in Table 1. Notably, group MU-1 demonstrated a significantly higher failure load value compared to the other study groups. No significant difference was observed between groups Ti-1 and MU-2, while significant differences were found between the remaining groups (*p*=0.001; *p* < 0.05).

**Figure 3:**
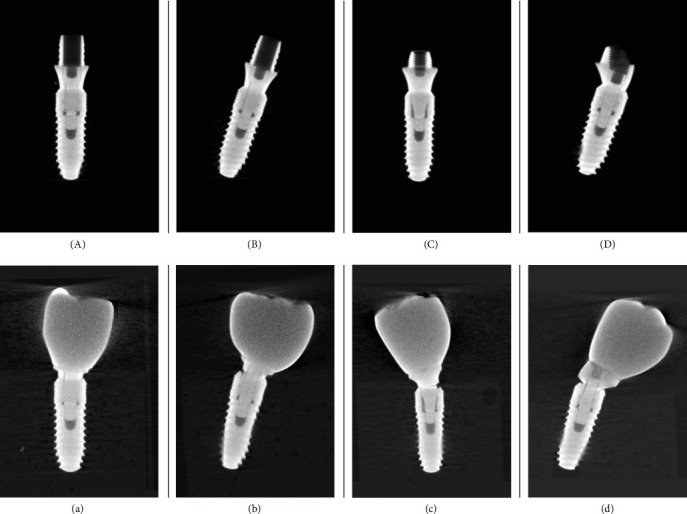
Micro-CT images of unloaded samples and micro-CT images showing representative screw bending of the loaded study samples. (A, a) Group Ti-1 control sample and group Ti-1 study sample; (B, b) group Ti-2 control sample and group Ti-2 study sample; (C, c) group MU-1 control sample and group MU-1 study sample; and (D, d) group MU-2 control sample and group MU-2 study sample.

Following quasi-static loading, the failure modes of prosthetic abutments varied between groups, with each specimen observed individually. No fractures of the implants or restorations, crown deformations, or adhesive failures were detected in any specimens. After removing the crowns, prosthetic components were examined, and visual inspection revealed bending in the Ti-base screws, multi-unit abutment necks, and screws. Additionally, distortions of the implant necks were observed in some specimens. The failure modes are presented in Table 2. Postloading micro-CT images indicated bending in the screws, with a significant angular difference between the control (unloaded) specimens and study specimens, which exhibited pronounced bending after loading. The variations in screw bending compared to unloaded screws are shown in Figure 3.

**Figure 4:**
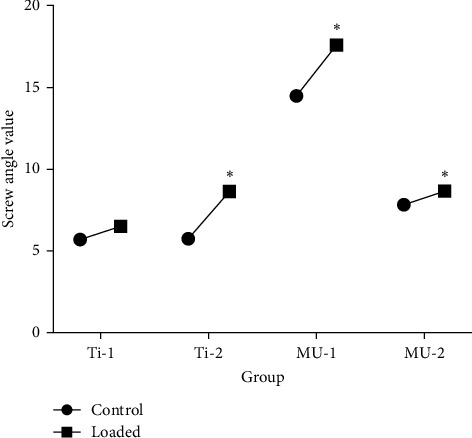
Screw bending value of the study groups. *⁣*^*∗*^ Represents statistical significance difference was observed within the control group and study group.

For statistical analysis, specimens in which screw bending was not distinctly observable were excluded from the final evaluation, and changes in bending values were assessed. To precisely measure deformation extent, bent screws were compared to their corresponding unloaded controls within each experimental group. Descriptive statistics derived from the micro-CT analysis are provided in Table 3, a statistically significant angular deviation in the abutment screws was observed in groups Ti-2, MU-1, and MU-2 relative to their control counterparts (*p*=0.001; *p* < 0.05). Conversely, the angular variation in group Ti-1 abutment screws did not exhibit a statistically significant difference from its control group. Detailed parametric data are depicted in Figure 4.

**Figure 5:**
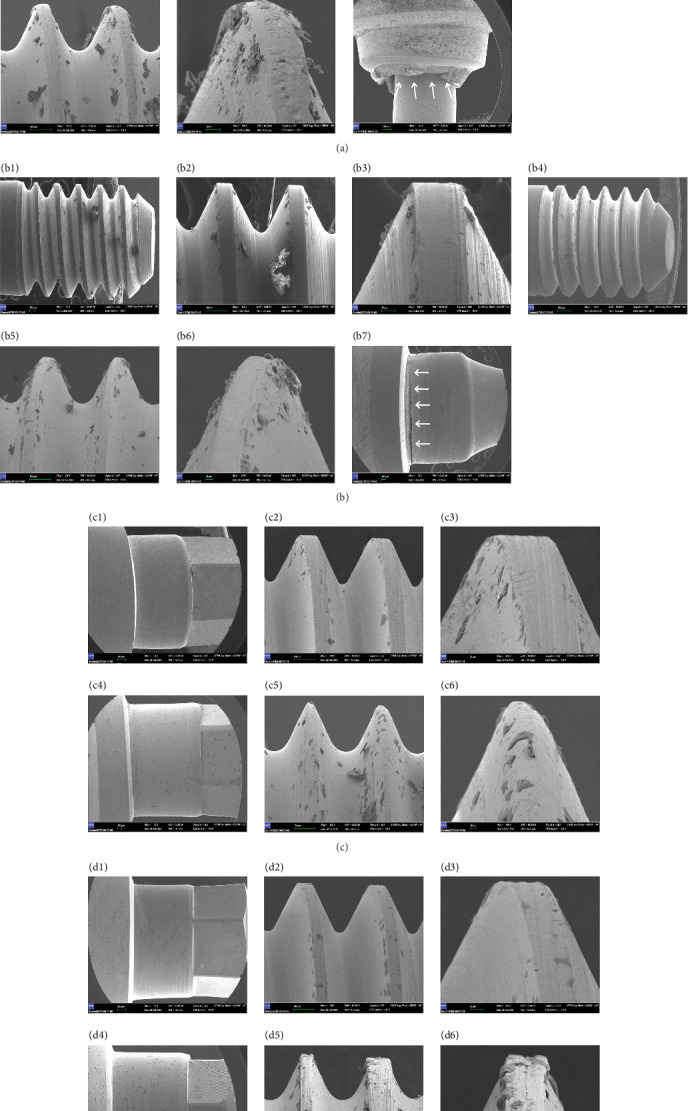
(a) Representative SEM images of the Ti-base abutment screw (a1–a3) before and (a4–a6) after quasi-static loading. Significant surface rounding of the threads is seen at 500x magnification. (a7) The fractured sample of Ti-base abutment with a straight implant (group Ti-1:1) after quasi-static loading. A buccal view of a fractured Ti-base abutment at the neck revealed an irregular fracture line (magnification ×30). The hackle lines (arrows) indicate the direction of crack propagation, and there are also deformations on the surface of the abutment in the inner portion that fits over the implant platform. (b) Representative SEM images of multi-unit abutment screws (b1–b3) before and (b4–b6) after quasi-static loading. (b7) A view of a crack line in the area of the neck of the multi-unit abutment (arrows), indicating a stress bearing area in the tested specimen of the straight multi-unit abutment (group MU-1:22). (c, d) Representative SEM images showing an overview of the angled Ti-base and angled multi-unit abutments,and increasing magnifications of the area between the threads. (c) Group Ti-2 abutment and abutment screw before (c1–c3) and after (c4–c6) quasi-static loading. (d) Group MU-2 abutment and abutment screw before (d1–d3) and after (d4–d6) quasi-static loading.

SEM imaging elucidated substantial structural modifications within the abutments of groups Ti-2 and MU-2, with notable mechanical damage localized on the abutment necks of groups Ti-1 and MU-1, attributable to stress-induced effects of static loading. Structural deformations, material wear, and plastic deformation were identified within the abutment screws across all groups (Ti-1, Ti-2, MU-1, and MU-2). SEM analysis of the screw threads demonstrated minor particulate debris and surface irregularities throughout. Following static loading, the screw threads displayed pronounced wear effects, including signs of abrasive wear, plastic deformation, and surface scoring, with material loss and kneading observed along thread faces. Importantly, no interthread cracking was detected (Figure 5).

## 4. Discussion

This in vitro study evaluated the performance of two different abutment types under quasi-static loading conditions, aiming to assess the maximum force resistance of multi-unit and Ti-base abutments used with implants placed in both straight and angled positions. The null hypothesis posited that no statistically significant differences would exist between Ti-base and multi-unit abutments regarding the prosthetic screw's mechanical resistance. However, significant differences were observed in the force values at which mechanical deformation occurred, leading to the rejection of the null hypothesis.

Previous studies report that maximum bite forces in the molar regions of healthy young adults range between 446 N and 1220 N [22]. The findings from this study indicate that the average observed failure load values exceed these clinical occlusal forces, suggesting that all examined implant crown structures are capable of withstanding maximal biting forces. However, it is important to note that individuals with implant-supported restorations might experience higher masticatory forces due to the absence of proprioception. This factor is particularly relevant in patients with bruxism, who may encounter intensified forces [23]. This in vitro study specifically tested the maximum resistance force of implant-supported fixed prosthesis abutments to exclude any influence from crown material. Additionally, the weakest part of the implant–abutment complex was evaluated.

The crowns used in this study were fabricated from Cr–Co alloy. Although full-metal crowns are not commonly used in clinical practice, this study was designed to observe prosthetic screw failures while eliminating any crown-material-related failures. Literature indicates that metal crowns have been extensively tested under both dynamic and static loading conditions in in vitro studies focusing on screw loosening and the abutment-implant connection [24, 25].

During chewing movements, the majority of forces applied are vertical, primarily involving compressive loads. Accordingly, the compressive strength of dental implants and abutments has been a significant focus of investigation within the literature [26, 27]. Although standardized protocols for quasi-static and cyclic loading compressive strength testing exist in various fields, including materials testing, these protocols are frequently adapted to accommodate the specific aims of each study [28]. In this study, all specimens were subjected to quasi-static loading to analyze the influence of implant angulation and abutment type on mechanical deformation capacity. The quasi-static loading protocol employed in the current study is in parallel with the one used by Nouh et al. [29]; however, the lack of repeated mechanical loading may account for the higher failure load values observed. The observed failure mode was plastic deformation of the titanium components, a distinct type of fatigue failure. Based on both experimental data and clinical experience, these results suggest that the specimens can reliably withstand functional masticatory forces.

The results further indicated that Ti-base abutments exhibited deformation at lower force values when paired with angled implants compared to straight implants. The literature supports that angled implants induce increased stress relative to those placed perpendicular to the occlusal plane [30]. In this study, abutments in angled implant groups (Ti-2 and MU-2) deformed under lower force values, indicating they experienced elevated stress levels. Specifically, one study found that angled abutments increase von Mises stress in the crestal cortical bone by 18% compared to straight abutments [31]. Another study reported a 26% increase in von Mises stress with a 25° angled abutment versus a straight abutment [32]. While these studies confirm elevated stress in the surrounding bone due to angled abutments, this does not necessarily indicate a risk of bone resorption. When angulation is required in implant placement, angled abutments provide a method for correcting the occlusal plane [33]. Some research even advocates for the strategic use of angled abutments to alleviate occlusal load-induced stress on angled implants. Findings from this study suggest that Ti-base abutments effectively reduce stress transmission to implants, abutments, and screws in angled implant configurations [34]. Furthermore, the angled abutment systems demonstrated deformation levels surpassing intraoral force thresholds, indicating their robustness against masticatory loads.

Our research findings indicate that the abutment screw is the weakest component within implant systems. Previous studies have corroborated that the abutment screw is particularly prone to stress concentration [35, 36]. After quasi-static loading, both multi-unit and Ti-base abutments withstood excessive forces; however, stress was localized on the most vulnerable component, resulting in bending. Consequently, bending of the abutment screws was observed across all groups. Notably, group MU-1 exhibited bending failure predominantly under higher loading forces, while group MU-2 displayed a bending pattern similar to that of group Ti-1. This study clearly demonstrates that multi-unit abutment systems offer greater resistance to applied forces compared to Ti-based abutment systems. Although bending was observed in the prosthetic screws across all groups, no fractures were noted.

Micro-CT imaging was utilized to perform 3D analyses of mechanical failures and component adaptation [37]. The micro-CT results revealed a significant increase in bending values for the screws in groups MU-1, MU-2, and Ti-2 compared to unloaded controls. Only the screws in group Ti-1 did not show a significant change in bending. Additionally, the abutment neck fracture observed in a group Ti-1 specimen suggests that stress accumulation in Ti-base abutments is concentrated in the abutment neck before transferring to the prosthetic screw.

The primary objective of this study was to examine the deformation of the abutment screw through SEM analysis, a method extensively validated as a reliable approach for such assessments [38]. The results indicated that all components subjected to compressive forces exhibited deformation. Specifically, both the threads of the abutment and prosthetic screws displayed surface deformation, with additional changes observed in the abutment neck and implant surface.

Thermal aging is a critical method for evaluating the effects of heat and humidity on dental restorations. Thermocycling, a widely recognized aging technique, subjects samples to alternating temperatures, inducing repeated expansion, contraction, and various stress factors that significantly impact in vitro study outcomes [39]. In this study, all specimens underwent in vitro thermal cycling of 5000 cycles using a specialized device, simulating approximately 6 months of intraoral exposure [21]. Additionally, resin cement was used for the cementation of crowns. The ISO 14801 test procedures do not specify a preferred type of cement, and a literature review revealed limited studies on the correlation between cement type and screw loosening. Nonetheless, several studies have favored resin cement for crown bonding [40, 41]. Following thermal aging and the load-to-failure test, no adhesive failures were observed in any specimens.

While numerous studies have examined screw loosening and abutment fractures in screw-retained restorations, there remains limited data comparing the mechanical performance of prosthetic screws in Ti-base versus multi-unit abutments, despite the increasing use of these restorations. This study aimed to directly compare the load-bearing capacities and failure modes of Ti-base and multi-unit abutments, considering both straight and angled implant placements. Another primary objective was to assess abutment performance under load in scenarios where ideal alignment is not achievable, often due to anatomical constraints such as the maxillary sinus, mandibular canal, mental foramen, nasal cavity, atrophic maxilla, narrow alveolar ridge, and crest morphology [42, 43]. To address these challenges, angled Ti-base or multi-unit abutments are often chosen in prosthetic treatments. This study evaluates prosthetic screws by using both straight and angled multi-unit and Ti-base abutments to adapt to varied implant orientations.

This study's limitations include a relatively small sample size, limiting the scope of statistical analysis; nonetheless, it highlights an important issue not previously documented in the literature. Future studies with larger samples are needed to confirm these findings. Additionally, this study used offset loading; however, oblique forces on the prosthesis could present greater risks. If an oblique load were applied, the titanium abutment might deform under lower forces or even fracture due to increased stress transfer to the screw. Further research should examine different loading angles for a more complete understanding.

Thermal cycling in this study aimed to replicate some intraoral conditions, but it did not fully mimic the dynamic oral environment, lacking factors like saliva, temperature changes, and actual occlusal forces. Future research could incorporate a masticatory simulator to more precisely evaluate mechanical impacts on prosthetic components. While laboratory simulations, including chewing and thermal cycling, can replicate aging effects to some degree, they do not fully capture the complexity of clinical conditions. Although this study accounted for certain limitations, further investigation is needed to address these aspects fully. Clinical trials are essential to verify these findings and understand any differences that might emerge under physiological conditions.

This study emphasizes that prefabricated screw-retained prosthetic abutments demonstrate the mechanical stability required for use in the posterior region, supporting their viability as a treatment option. The findings provide a solid foundation for further clinical studies with increased confidence.

## 5. Conclusion

As indicated by the study's results, the abutment screw is identified as the most vulnerable component in implant systems. Nonetheless, both the multi-unit and Ti-base abutment systems exhibited notably high load-to-failure values, demonstrating that all evaluated screw systems can withstand typical intraoral forces. The straight multi-unit abutments showed higher load-to-failure values compared to other groups, underscoring their reliability as a choice for clinical applications. However, abutment systems designed for angled implants deformed at lower force thresholds, indicating a variation in mechanical resistance related to implant angulation.

## Figures and Tables

**Table 1:** Descriptive statistics of the failure loads (*N*).

Groups	Failure load (*N*) Mean ± Ss	Min–max (median)	Confidence interval (%95)	*n*
Ti-base 1	2589.73 ± 420^a^	1990.73–3231.19 (2557.77)	2289.27–2890.17	10
Ti-base 2	1768.09 ± 359.16^b^	1339.24–2403.79 (1643.93)	1511.16–2025.01	10
Multi-unit 1	3318.79 ± 636.33^c^	2793.85–4764.7 (3074.77)	2863.59–3773.99	10
Multi-unit 2	2213.81 ± 459.44^a^	1242.51–2903.72 (2248.19)	1885.15–2542.47	10

*Note*: Kruskal–Wallis test. Different superscript lowercase letters indicate significant differences among groups.

**Table 2:** Overview of failure modes after quasi-static loading test.

Failure mode	Adhesive failure	Crown deformation	Implant deformation	Abutment fracture	Abutment bending	Screw fracture	Screw bending
Ti-base 1 (*n* = 10)	0	0	10	1	7	0	10
Ti-base 2 (*n* = 10)	0	0	10	0	7	0	10
Multi-unit 1 (*n* = 10)	0	0	10	0	8	0	10
Multi-unit 2 (*n* = 10)	0	0	10	0	5	0	10

**Table 3:** Descriptive statistics of the screw angle value.

Sample size (*n*)		Mean ± Ss	Min–max (median)	Confidence interval (%95)	*p*
Screw angle value					
Grup-1	7	6.53 ± 1.25^a^	5.52–8.84 (5.79)	5.37–7.68	0.315
Grup-1 control	10	5.69 ± 0.36^a^	5.09–6.11 (5.76)	5.43–5.94	—
Screw angle value					
Grup-2	9	8.62 ± 1.51^a^	6.2–9.96 (9.29)	7.12–9.45	**0.001** *⁣* ^*∗∗*^
Grup-2 control	10	5.74 ± 0.3^b^	5.17–6.11 (5.82)	5.52–5.96	—
Screw angle value					
Grup-3	10	17.58 ± 1.42^a^	15.9–20.71 (17.43)	16.56–18.59	**0.001** *⁣* ^*∗∗*^
Grup-3 control	10	14.46 ± 0.37^b^	13.82–14.87 (14.53)	14.20–14.73	—
Screw angle value					
Grup-4	9	8.65 ± 0.72^a^	7.857–9.782 (8.59)	8.10–9.20	**0.001** *⁣* ^*∗∗*^
Grup-4 control	10	7.81 ± 0.26^b^	7.455–8.306 (7.81)	7.63–7.99	—

*Note*: Different superscript lowercase letters indicate significant differences among groups. Bold indicates a statistically highly significant difference.

## Data Availability

The data that support the findings of this study are available from the corresponding author upon reasonable request.
